# Effective estimation of correct platelet counts in pseudothrombocytopenia using an alternative anticoagulant based on magnesium salt

**DOI:** 10.1111/bjh.12443

**Published:** 2013-06-29

**Authors:** Peter Schuff-Werner, Michael Steiner, Sebastian Fenger, Hans-Jürgen Gross, Alexa Bierlich, Katrin Dreissiger, Steffen Mannuß, Gabriele Siegert, Maximilian Bachem, Peter Kohlschein

**Affiliations:** 1 Institute of Clinical Chemistry and Laboratory Medicine, Rostock University Medical CentreRostock, Germany; 2 Medical Laboratory RostockRostock, Germany; 3 Core Facility of Clinical Chemistry, University Medical Centre UlmUlm, Germany; 4 Institute of Clinical Chemistry and Laboratory Medicine, University Hospital “Carl Gustav Carus”, University of Dresden Medical FacultyDresden, Germany

**Keywords:** platelet aggregation, pseudothrombocytopenia, anticoagulation, magnesium, EDTA, citrate

## Abstract

Pseudothrombocytopenia remains a challenge in the haematological laboratory. The pre-analytical problem that platelets tend to easily aggregate *in vitro*, giving rise to lower platelet counts, has been known since ethylenediamine-tetra acetic acid EDTA and automated platelet counting procedures were introduced in the haematological laboratory. Different approaches to avoid the time and temperature dependent *in vitro* aggregation of platelets in the presence of EDTA were tested, but none of them proved optimal for routine purposes. Patients with unexpectedly low platelet counts or flagged for suspected aggregates, were selected and smears were examined for platelet aggregates. In these cases patients were asked to consent to the drawing of an additional sample of blood anti-coagulated with a magnesium additive. Magnesium was used in the beginning of the last century as anticoagulant for microscopic platelet counts. Using this approach, we documented 44 patients with pseudothrombocytopenia. In all cases, platelet counts were markedly higher in samples anti-coagulated with the magnesium containing anticoagulant when compared to EDTA-anticoagulated blood samples. We conclude that in patients with known or suspected pseudothrombocytopenia the magnesium-anticoagulant blood samples may be recommended for platelet counting.

Enumeration and differentiation of circulating blood cells is performed in the haematological laboratory using appropriately anticoagulated whole blood samples. With the introduction of commercially available blood-collecting systems, salts of ethylenediamine-tetra acetic acid (EDTA) either as di-sodium, di-potassium or tri-potassium salt, are the anticoagulants of choice, and recommended by the International Council for Standardization in Haematology (1993). EDTA chelates divalent cations including calcium, thus avoiding coagulation and stabilizing the sample for subsequent haematological analysis (Banfi *et al*, 2007).

The use of EDTA is generally accepted to be safe and reliable for obtaining full blood counts. In addition, EDTA salts are compatible with the standard staining protocols for blood smears. One drawback of EDTA salts is a time-dependent osmotic effect, leading to increasing mean cellular volume (MCV) of red blood cells (RBC). If EDTA-anticoagulated samples are not analysed within 24 h, this might lead to misinterpretation, e.g. false high haematocrit values (Robinson *et al*, 2004).

Much more critical and of proven diagnostic relevance, is the rare occurrence of false low platelet counts in the presence of EDTA, so-called pseudothrombocytopenia (PTCP) (Shreiner & Bell, 1973). Platelet aggregates may be misclassified by haematological analysers as leucocytes and therefore a false high white blood cell (WBC) count may result and escape interpretation as pseudoleucocytosis (Solanki & Blackburn, 1983). PTCP has been shown to be a time- and temperature-dependent phenomenon. Therefore, direct sampling and immediate analysis results in higher platelet counts compared to those obtained after delay. PTCP and recommendations for its prevention were recently reviewed in detail (Lippi & Plebani, 2012).

Although EDTA was introduced in the haematological laboratory in the 1950s (Proesche, 1951), the first report of EDTA-induced platelet aggregation was not reported until 1969 (Gowland *et al*, 1969). A number of studies notwithstanding, the pathophysiology of abnormal platelet aggregation in the presence of EDTA is not fully understood. In selected cases of EDTA-induced PTCP, blood was recollected using citrate or heparin, but *in vitro* aggregation was still present (Schrezenmeier *et al*, 1995).

Before the introduction of EDTA salts, magnesium salts were used as an anticoagulant for sampling capillary blood for microscopic platelet enumeration, as communicated in a widely respected laboratory handbook in the early 1930s (Lenhartz & Meyer, 1934). However, the anti-aggregatory properties of magnesium were forgotten after the introduction of EDTA as an anticoagulant in the haematological laboratory.

These historical accounts inspired us to start a multicentre study using manufactured blood sampling tubes anticoagulated with magnesium sulfate. The aim of our observational study was to compare platelet counts in patients showing EDTA-induced aggregation indicated by aggregation flags and consecutive PTCP, as counted in EDTA- and magnesium sulfate-anticoagulated samples.

Furthermore, citrate anticoagulated samples were examined for aggregation in one of the participating laboratories.

## Patients and methods

This study was performed in three routine haematology laboratories (Rostock, Ulm, Dresden, Germany) after approval by the institutional review board of the University of Rostock.

EDTA-anticoagulated whole blood samples for routine haematological analysis were selected for this study when flagged as suspicious for platelet aggregates (even in the case of platelet counts still appearing to be within the normal range). If platelet aggregates were confirmed by microscopic examination of blood smears, the patient was asked for written consent to obtain additional blood samples using collecting tubes anticoagulated with EDTA or magnesium sulfate or, in some cases, sodium citrate samples measured in parallel.

At the beginning of the study, sampling tubes anticoagulated with magnesium sulfate (final concentration 33·8 μmol MgSO_4_) were provided by the manufacturer (Sarstedt, Nürmbrecht, Germany). Later during the study period these tubes became commercially available (Thromboexact™ Monovette, Sarstedt, Nürmbrecht, Germany).

Platelets were counted by automated routine haematological analysers: two of the participating laboratories used a Coulter LH 750 system (Beckman Coulter, Krefeld, Germany) and one laboratory was equipped with a Sysmex XE 5000 system (Sysmex, Norderstedt, Germany). Blood smears were prepared and May-Grünwald/Giemsa stained according to standard operating procedures (Pappenheim, 1908). Peripheral blood films were analysed and documented by computer-assisted microscopic differentiation (Diffmaster™, Sysmex). One of the participating laboratories modified the protocol by analysing platelet counts in citrate-anticoagulated blood samples (*n* = 10) after mathematical correction for dilution by the anticoagulant.

To demonstrate the anti-aggregating effect of the magnesium-based anticoagulation, platelet aggregation studies were performed in blood samples from healthy donors collected by the Thromboexact™ (TE) and the standard hirudin-containing tube (S-Monovette™ Hirudin, Sarstedt, Nürmbrecht, Germany). Aggregation studies were performed using a Multiplate™ analyser (Dynabyte Medical, Munich, Germany). Aggregation was induced by adenosine diphosphate (ADP test) or by arachidonic acid (ASPI test) and is expressed as area under the curve (AUC). The normal aggregation elicited by ADP ranges from 53 to 122 units (u), and for ASPI from 75 to 136 u, respectively. The analyser and the procedures were previously described in detail (Toth *et al*, 2006).

### Statistical analysis

SPSS version 16·0 (IBM, NY, USA) was used for statistical analysis. Descriptive statistics [including mean, standard deviation (SD) and standard error of mean (SEM)] were calculated to characterize the study population. The normal distribution of the complete blood count (CBC) parameters was checked using the Kolmogorow–Smirnow test. All comparisons for statistical significance between two anticoagulants were performed using the paired *t* test. Statistical significance was achieved if *P* < 0·05.

## Results

In three different University-based haematology laboratories a total of 44 patients (26 females and 18 males) aged between 18 to 72 years, were included in this study. Clinical diagnoses included coronary heart disease (*n* = 12), acute upper respiratory tract infections (*n* = 5), diabetes mellitus or acute trauma or postoperative care (*n* = 17). No specific diagnoses were obtained for the remaining patients. The criteria for selecting PTCP patients were unexpectedly low platelet counts and/or positive flagging for platelet aggregates.

**Fig 1:**
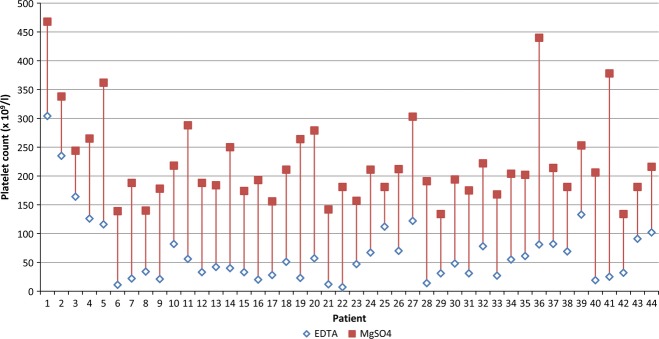
Platelet counts in 44 patients with EDTA-induced PTCP (open rhombus) corrected by parallel measurement in magnesium-anticoagulated blood samples (filled squares). The difference is statistically significant (paired *t*-test). In three individuals the analyser flagged the platelet count for aggregation although the values appeared to be in the normal range. This is due to the fact that PTCP is time-dependent and platelets were measured relatively early after blood sampling.

Platelet counts in samples flagged for ‘platelet aggregates’ although anticoagulated with EDTA ranged from 7 × 10^9^/l to 304 × 10^9^/l whereas in magnesium-anticoagulated samples from the same individuals the platelet counts ranged from 134 × 10^9^/l to 468 × 10^9^/l (Fig 1).

The mean platelet count in EDTA-anticoagulated blood of individuals with PTCP was 66 × 10^9^/l whereas the mean platelet count in magnesium-anticoagulated tubes was 223 × 10^9^/l. The mean absolute increase of platelets in samples anticoagulated with magnesium sulphate was 154 × 10^9^/l (+230%) ranging from 69 × 10^9^/l (+162%) to 285 × 10^9^/l (+475%). In one individual, the initial platelet count was 7 × 10^9^/l in the presence of EDTA; this increased to 181 × 10^9^/l when measured in parallel using TE.

The study protocol did not schedule the use of citrated blood samples in parallel; nevertheless in 10 patients such data were available (see Table I). In four patients the platelet count was comparable to those obtained in MgSO_4_; five out of six patients showing lower platelet counts in citrate samples were flagged for aggregates. The count differences to the results achieved with magnesium anticoagulant samples ranged between 92 and 181 × 10^9^/l.

**Table I:** Platelet counts in blood samples from 10 individuals with pseudothrombocytopenia anticoagulated with ethylenediamine-tetra acetic acid (EDTA), citrate or MgSO_4_

	Platelet count (×10^9^/I)		
Patient	EDTA	Citrate	MgSO_4_
4	126^*^	266	265
5	57^*^	172^*^	279
20	25^*^	313	378
21	116^*^	356	362
23	78^*^	213	222
30	12^*^	20^*^	142
32	81^*^	269^*^	440
36	48	102	194
41	47^*^	66^*^	157
44	102	222	216

The samples flagged by the Sysmex haematology analyser are identified by an asterisk.

**Fig 2:**
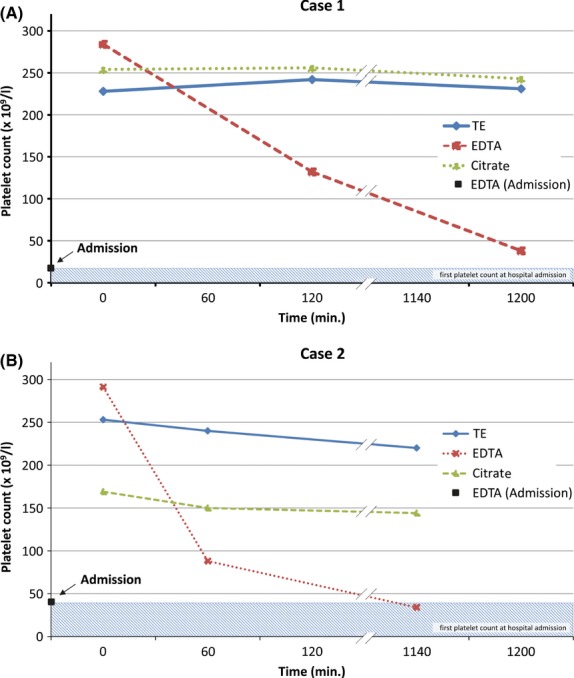
Time-dependent progression of anticoagulant-induced pseudothrombocytopenia (PTCP). Both cases were documented independently from the primary study. The black square and the hatched area indicate the first platelet count performed in an EDTA-anticoagulated blood sample drawn at admission and measured after arrival of the sample at the routine haematological laboratory. The red line (cross symbols) represents the time-dependent decrease of platelets in the EDTA-anticoagulated sample; the green line indicates platelet counts in citrate anticoagulated blood and the blue line (rhombus symbol) demonstrates platelet counts in magnesium-anticoagulated blood. EDTA, ethylenediamine-tetra acetic acid; TE, Thromboexact™.

In two patients we documented the influence of time and anticoagulant on the phenomenon of PCTP in more detail. On receipt of the EDTA-anticoagulated samples in the laboratory, platelet counts were low (17 × 10^9^/l and 35 × 10^9^/l). Thrombocytopenia, unexpected for clinical reasons but suggested by platelet aggregate flag, led us ask the patient to consent for additional sampling of blood anticoagulated with EDTA, citrate, and magnesium sulfate, respectively. The platelet counts were immediately performed in all samples, yielding numbers within the reference interval of 284/291 × 10^9^/l (EDTA), 254/169 × 10^9^/l (citrate), and 228/253 × 10^9^/l (TE). With progressively elapsing time between blood collection and platelet enumeration, platelet counts decreased dramatically in the EDTA sample, in contrast to the samples anticoagulated with citrate or magnesium (Fig 2A). In the second patient the platelet count at admission was also low, platelet counts in consecutively collected new samples (anticoagulated by EDTA, citrate und magnesium sulphate) are depicted in Fig 2B. After one and two hours of storage at room temperature the platelet counts decreased continuously in the EDTA samples of both patients. Platelet counts also decreased in the citrate sample from one patient. The number of platelets in the TE sample remained stable for up to 20 h (Fig 2A, B).

**Fig 3:**
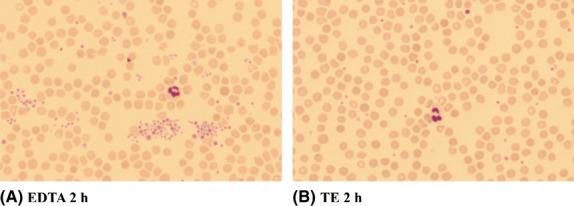
Platelet aggregation in a blood smear from a patient with EDTA-induced pseudothrombocytopenia (left) as compared to a smear prepared from magnesium-anticoagulated blood from the same patient (right). EDTA, ethylenediamine-tetra acetic acid; TE, Thromboexact™.

Platelet aggregation as a function of time in EDTA samples was documented in stained blood smears (Fig 3). In contrast, platelet aggregation could not be detected in magnesium-anticoagulated samples.

**Fig 4:**
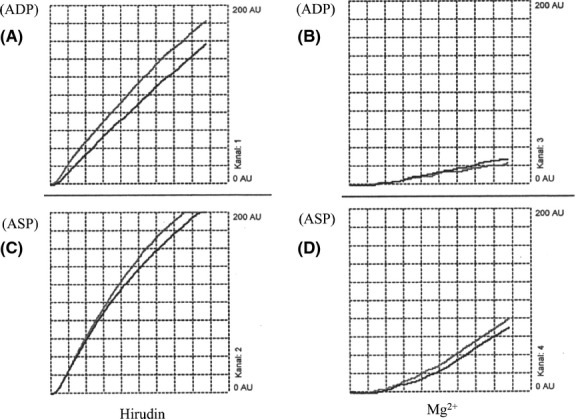
Multiplate^R^ platelet function analysis. Platelet aggregation induced by adenosine diphosphate (ADP) (A, B) or arachidonic acid (ASP) (C, D) in whole blood samples from a healthy donor with normal platelet count (320 × 10^9^/l). Blood was drawn into collection tubes containing hirudin (A, C) or magnesium (B, D). The areas under the curve (AUC) are expressed by arbitrary units (u). The ADP-induced aggregation (hirudin tubes) gave an AUC of 81 u (53–122 u), the ASP-induced aggregation gave an AUC of 110 u (75–136 u). The corresponding results in blood samples collected into Thromboexact™ tubes were 10 u and 25 u, respectively.

We further compared platelet aggregation in healthy volunteers using the Multiplate™ analyser. Aggregation induced by ADP or arachidonic acid was effectively inhibited when platelets were collected in TE (example shown in Fig 4). The AUC values (u) as a measure of aggregation were suppressed by approximately 88% and 77% when compared to platelets collected in hirudin-anticoagulated tubes. This suppression of platelet aggregation was confirmed in three further experiments with similar results (data not shown).

To prove that magnesium-anticoagulated blood samples can also be used for the determination of other routine haematological parameters we compared the automated WBC count and differentiation as well as the quality of blood smears for microscopic WBC differentiation in EDTA- and magnesium-anticoagulated samples (Table I and III). We compared erythrocytes, leucocytes and differential counts in EDTA- and magnesium-anticoagulated samples (Table II and III). Erythrocytes, leucocytes and differential counts were comparable. The statistical comparison showed no significant difference between both anticoagulants, although the number of platelets was slightly lower in magnesium-anticoagulated blood.

**Table II:** Comparison of absolute complete blood count (CBC) in blood samples from non-pseudothrombocytopenic individuals anticoagulated either with ethylenediamine-tetra acetic acid (EDTA) or MgSO_4_. Statistical analysis showed no significant difference in CBC between both anticoagulants, although the number of platelets is slightly lower in MgSO_4_ anticoagulated blood. The compared cell counts are strongly correlated as expressed by correlation coefficient >0·9

	Erythrocytes		Leucocytes		Platelets		Neutrophils		Lymphocytes		Monocytes		Eosinophils		Basophils	
Patient	EDTA	MgSO_4_	EDTA	MgSO_4_	EDTA	MgSO_4_	EDTA	MgSO_4_	EDTA	MgSO_4_	EDTA	MgSO_4_	EDTA	MgSO_4_	EDTA	MgSO_4_
N1	3·07	3·04	3·11	3·03	198·00	152·00	1·77	1·69	1·03	1·06	0·22	0·17	0·08	0·09	0·01	0·02
N2	4·05	4·06	10·01	9·82	378·00	325·00	7·41	7·42	1·72	1·58	0·52	0·48	0·35	0·33	0·01	0·02
N3	2·94	2·97	5·07	5·02	206·00	186·00	3·48	3·44	1·08	1·10	0·40	0·37	0·08	0·10	0·03	0·03
N4	3·44	3·45	9·29	8·63	185·00	170·00	7·52	6·96	1·00	0·96	0·50	0·48	0·25	0·19	0·02	0·06
N5	4·30	4·85	8·40	8·05	178·00	156·00	5·03	4·79	2·35	2·39	0·69	0·79	0·05	0·06	0·02	0·02
N6	4·90	4·74	9·76	9·74	116·00	107·00	8·60	8·58	0·44	0·46	0·63	0·63	0·09	0·07	0·00	0·02
N7	4·41	4·74	5·68	5·67	266·00	234·00	2·99	2·93	2·01	1·96	0·51	0·62	0·15	0·14	0·02	0·01
N8	4·63	4·71	8·52	8·10	352·00	279·00	3·25	3·20	3·76	3·55	0·73	0·61	0·70	0·65	0·08	0·12
N9	4·65	4·64	5·87	5·61	181·00	155·00	3·04	2·91	1·66	1·68	0·62	0·49	0·52	0·51	0·03	0·03
N10	3·86	4·44	6·06	5·76	181·00	168·00	3·53	3·33	1·91	1·90	0·37	0·28	0·23	0·22	0·02	0·02
N11	4·29	4·42	8·35	7·58	299·00	223·00	5·37	5·00	1·35	1·04	1·14	1·18	0·45	0·32	0·45	0·04
N12	3·98	4·39	5·12	4·77	405·00	322·00	3·61	3·40	0·90	0·83	0·52	0·44	0·07	0·07	0·02	0·03
N13	4·42	4·34	18·74	17·58	306·00	262·00	14·38	13·68	1·90	0·68	2·33	2·11	0·12	0·09	0·01	0·02
N14	3·03	3·90	12·97	12·50	378·00	326·00	10·22	9·85	1·29	1·38	1·29	1·10	0·15	0·14	0·02	0·03
N15	2·69	3·86	10·57	10·48	389·00	355·00	8·00	8·00	1·75	1·63	0·41	0·46	0·31	0·30	0·10	0·08
N16	3·84	3·84	11·98	11·57	225·00	205·00	9·74	9·42	1·17	1·06	0·78	0·79	0·28	0·28	0·01	0·03
N17	5·07	5·05	8·59	8·77	215·00	186·00	5·74	5·74	1·70	1·91	0·78	0·70	0·32	0·37	0·04	0·05
N18	5·22	5·26	7·66	7·85	195·00	177·00	5·61	5·71	1·55	1·57	0·42	0·50	0·07	0·06	0·01	0·01
N19	5·05	5·00	5·12	5·11	175·00	150·00	2·54	2·45	2·05	2·11	0·40	0·42	0·10	0·11	0·03	0·02
N20	5·01	5·06	5·69	5·21	299·00	258·00	2·51	2·46	2·58	2·27	0·47	0·38	0·10	0·07	0·03	0·03
N21	5·17	5·17	6·66	6·88	271·00	226·00	3·43	3·51	2·34	2·34	0·60	0·69	0·26	0·31	0·03	0·03
Mean	4·19	4·38	8·25	7·99	257·05	220·10	5·61	5·45	1·69	1·59	0·68	0·65	0·23	0·21	0·05	0·03
SEM	0·17	0·15	0·76	0·72	18·65	15·28	0·70	0·68	0·16	0·16	0·10	0·09	0·04	0·04	0·02	0·01
r	0·90		0·99		0·98		0·99		0·92		0·98		0·98		0·25	

SEM, standard error of the mean.

**Table III:** Comparison of absolute complete blood count (CBC) in blood samples from pseudothrombocytopenic (PTCP) individuals anticoagulated either with ethylenediamine-tetra acetic acid (EDTA) or MgSO_4_ collected at the same time. The CBC were similar except for platelet counts, due to EDTA-induced PTCP, which were corrected by MgSO_4_

	Erythrocytes		Leucocytes		Platelets		Neutrophils	
Patient	EDTA	MgSO_4_	EDTA	MgSO_4_	EDTA	MgSO_4_	EDTA	MgSO_4_
23	4·63	4·38	17·16	14·23	102	209	13·31	11·41
30	4·93	4·99	10·05	10·24	47	157	5·66	5·60
44	4·81	4·81	5·74	5·86	48	194	4·09	4·21

Patient	Lymphocytes		Monocytes		Eosinophils		Basophils	
	EDTA	MgSO_4_	EDTA	MgSO_4_	EDTA	MgSO_4_	EDTA	MgSO_4_
23	2·21	1·72	1·62	1·09	0·00	0·00	0·02	0·01
30	3·14	3·37	1·00	1·02	0·22	0·21	0·03	0·04
44	1·19	1·18	0·39	0·4	0·05	0·05	0·02	0·02

The quality of blood smears prepared from EDTA- and magnesium-anticoagulated blood samples was comparable and the results of microscopic WBC differentiation, documented by computer-assisted microscopy, showed no significant deviations (data not shown).

## Discussion

The present study aimed to demonstrate that the platelet-inhibiting potential of magnesium sulfate can effectively prevent platelet aggregation in whole blood samples from patients showing PTCP in the presence of EDTA.

Platelets are not fully stabilized in EDTA-anticoagulated blood samples. They undergo changes in shape and size and finally acquire a more spheroid form than their native discoid shape, thus leading to time-dependent changes of the mean platelet volume (MPV) (McShine *et al*, 1990; Banfi *et al*, 2007). This is probably the main reason for the conflicting database concerning the diagnostic value of MPV analysis (Thompson *et al*, 1983; Martin & Trowbridge, 1990). Therefore, alternative anticoagulants were repeatedly proposed to be used for MPV analysis, including adenosine/citrate/dextrose and sodium EDTA (Thompson *et al*, 1983), sodium citrate and prostaglandin E1 (Trowbridge *et al*, 1985), citrate/theophyllin/adenosine/dextrose and pyridoxal phosphate (Lippi *et al*, 1990; Ohnuma *et al*, 1988).

Recently it has been proposed that EDTA-induced platelet clumps can be dissociated by a mixture of calcium chloride for re-association of glycoprotein (GP) IIb/IIIa complex and sodium heparin for maintaining anticoagulation to correctly estimate platelet counts (Chae *et al*, 2012). The addition of an aminoglycoside antibiotic (e.g. kanamycin) has similarly been used to count platelets in cases of PTCP (Sakurai *et al*, 1997; Hae *et al*, 2002). The mechanism of this effect is not clear but it might be explained by sodium-citrate, which is an additive for stabilizing the drug preparation.

What all these anticoagulant cocktails have in common is that they are unstable and that their individual preparation is expensive. For MPV analysis, citrate anticoagulation as used for RBC sedimentatio seems more practicable and reliable compared to EDTA (von Stein, 2004). However, cell counts need to be corrected due to the dilution effect of added citrate solution. Practicability, stability and cost issues continue to call for alternative new anticoagulants for platelet analysis.

In individuals without EDTA-induced PTCP the platelet counts were regularly lower in samples anti-coagulated with magnesium-sulfate. This phenomenon is obviously due to the fact that the MPV is higher in EDTA anti-coagulated blood (10·6 fl) as compared to magnesium anti-coagulated blood (9·6 fl). The time-dependent swelling of cells in EDTA samples is known from the literature (McShine *et al*, 1990; Macey *et al*, 2002).

Automated haematological analysers are calibrated with EDTA samples; this might explain that the lower MPV of platelets in magnesium samples leads to the loss of platelets with an MPV lower than the values set as ‘lower gate’ for platelet counting.

The phenomenon of PTCP is usually observed in the presence of EDTA as anticoagulant (Mant *et al*, 1975). It is known from the literature that alternative anticoagulants, such as citrate or heparin, may also lead to *in vitro* platelet aggregation (McShine *et al*, 1990; Schrezenmeier *et al*, 1995). In the present study we also documented PTCP in samples anticoagulated by citrate and in one case also in the presence of heparin (data not shown). Although rare, the observation of spontaneous aggregation in the presence of different anticoagulants raises the question of whether this might be related to different underlying mechanisms of action. Schrezenmeier *et al* (1995) proposed that the phenomenon of *in vitro* platelet aggregation should be collectively called ‘anticoagulant-induced PTCP’.

The underlying mechanism of the time-dependant aggregate formation is at present unknown. It has been postulated that cold-reactive anti-platelet antibodies, mainly of the IgG class, are directed against a hidden epitope of the platelet GPIIa/GPIII receptor complex. This epitope becomes accessible because of conformational changes of the receptor due to the calcium complexing effect of EDTA (Fitzgerald & Phillips, 1985).

There are only a few reliable data on the incidence of EDTA-induced PTCP. The overall prevalence is estimated to be approximately 0·10%, with numbers being slightly higher in ill or hospitalized patients as compared to outpatients (Payne & Pierre, 1984; Vicari *et al*, 1988; Silvestri *et al*, 1995; Bartels *et al*, 1997). In highly selected patients with thrombocytopenia of unknown origin, the prevalence increases to between 1·25% and 15% (Manthorpe *et al*, 1981; Silvestri *et al*, 1995). Females seem to have a slightly higher incidence than males (ratio 3:2) as reported earlier (Püllen & Briehl, 1994). The occurrence of PTCP is not associated with any specific disease entity and has been described in healthy persons (Sweeney *et al*, 1995). Therefore, it was referred to as a ‘laboratory disease’ (Gschwandtner *et al*, 1997).

If PTCP is suspected in the haematology laboratory because of an analyser flag and/or for clinical issues (unexpected low platelet count not matching clinical presentation), a peripheral blood smear easily documents platelet aggregates to confirm the diagnosis. A new blood sample, anticoagulated with citrate is usually collected and rapidly transported to the laboratory for automated platelet counting without delay. Results need to be corrected for the citrate-based dilution effect. Nevertheless, in 15–20% of EDTA-induced PTCP platelets may also aggregate in the presence of citrate (Bizarro, 1995), rendering this approach limited for practical reasons.

Before EDTA was introduced as the anticoagulant of choice for haematology testing, magnesium salts were used as anticoagulants for sampling capillary blood for platelet enumeration (Lenhartz & Meyer, 1934). Later, magnesium was used as systemic anticoagulant in patients suffering from coronary heart disease (Gries *et al*, 1999; Shecter *et al*, 1999). Although this aggregation-inhibiting effect has been known for decades, the true underlying mechanisms remain unknown.

Magnesium has been labelled ‘nature’s calcium antagonist’ due to its ability to reduce the thrombin-stimulated calcium influx into platelets, which is one of the initial steps of platelet aggregation. *In vitro* it was also shown that the release of ß-thromboglobulin and thromboxane B2 from platelets is reduced with increasing magnesium concentrations (Hwang *et al*, 1992). Furthermore, magnesium may induce membrane fluidity changes, thus interfering with fibrinogen binding to the GPIIb/IIIa complex. By this action magnesium triggers the formation of AMP, ultimately resulting in inhibition of P 47 phosphorylation and intracellular calcium mobilization (Sheu *et al*, 2002). Our investigations on platelet aggregability (Fig 4) confirm the observation that magnesium inhibits platelet aggregation stimulated either by strong (collagen, thrombin; data not shown) or by weak agonists (ADP, arachidonic acid). Therefore it is surprizing that magnesium never became a candidate blood anticoagulant to be used for blood cell counting.

There are very few publications dealing with the usefulness of magnesium as anticoagulant in the laboratory (Kondo *et al*, 2002). The only publication on its effect in pseudothrombocytopenia was published in Japanese and probably reached only a smaller section of the scientific community (Nakamoto *et al*, 1986). Using an automated blood cell analyser (Sysmex SE 2100), the authors investigated the time-dependent effect of magnesium- and EDTA-anticoagulation on blood cell enumeration and automated WBC differentiation in patients with PTCP. They reported that magnesium sulfate normalized the platelet counts without interfering with other blood cell counts or differentiation results. These data find full confirmation in our present investigation.

If cases of PTCP are not being identified in the laboratory and/or for clinical reasons, the falsely low platelet count may trigger unnecessary, expensive and even invasive diagnostic or therapeutic procedures (Nilsson & Norberg, 1986; Yamada *et al*, 2008). To avoid this and to enable a safe and practical alternative, we recommend the use of magnesium sulphate anticoagulated blood samples for platelet counting if blood counts from EDTA samples do not match clinical expectations and/or are being flagged as suggestive of platelet aggregates by the automated haematology analyser.

## Conflict of interest

The authors PSW and MS were supported in part by a research grant from Sarstedt (Nürmbrecht, Germany). PSW received lecture honoraria by B. Braun-Melsungen (Melsungen, Germany), Roche (Mannheim, Germany), Beckmann-Coulter (Krefeld, Germany) and Sarstedt (Nürmbrecht, Germany).
